# Handgrip strength and body mass index exhibit good predictive value for sarcopenia in patients on peritoneal dialysis

**DOI:** 10.3389/fnut.2024.1470669

**Published:** 2024-12-13

**Authors:** Hongyan Li, Yuanhua Zheng, Yuanyuan Zhang, Xiaotian Zhang, Wei Luo, Weiyi Zhu, Yaqing Zhang

**Affiliations:** ^1^School of Nursing, Shanghai Jiao Tong University School of Medicine, Shanghai, China; ^2^School of Nursing, Jiangxi Medical College, Nanchang University, Nanchang, China; ^3^Peritoneal Dialysis Center, The First Affiliated Hospital, Nanchang University, Nanchang, China; ^4^Urology Center, Shanghai Jiao Tong University School of Medicine Affiliated General Hospital, Shanghai, China; ^5^Department of Nursing, Jiangxi Provincial People's Hospital, Nanchang, Jiangxi, China; ^6^Department of Nursing, Ruijin Hospital, Shanghai Jiao Tong University School of Medicine, Shanghai, China

**Keywords:** body mass index, handgrip strength, nomogram, peritoneal dialysis, risk factors, sarcopenia

## Abstract

**Aim:**

The diagnosis of sarcopenia in patients on peritoneal dialysis (PD) in clinics is limited owing to its relatively complicated process and the need for expensive assessment equipment. This study aimed to develop and validate sex-specific nomogram models based on body mass index (BMI), handgrip strength, and other routine follow-up examination indicators to predict sarcopenia in patients on PD.

**Methods:**

From March 2023 to February 2024, 699 eligible patients were recruited from the PD centers of two tertiary hospitals in southeastern China. Routine follow-up examination indicators such as age, BMI, biochemical indicators, dialysis adequacy, handgrip strength, and five-repetition sit-to-stand test, were used as potential predictive variables. Multivariate logistic regression analyses were used to separately determine the predictive factors for men and women. Nomogram models were constructed based on the results of the multivariate analyses, which were internally validated using a bootstrap re-sampling method (*n* = 2000). Predictive performance was validated using a receiver operating characteristic (ROC) curve.

**Results:**

The prevalence of sarcopenia in Chinese patients on PD was 13.92%. The nomogram models based on multivariate analyses revealed both handgrip strength and BMI as independent predictors of sarcopenia in men and women on PD. The bootstrap-corrected area under the ROC curves of the models was 0.924 (95% CI: 0.888–0.959) and 0.936 (95% CI, 0.906–0.966) for men and women, respectively. The calibration curves of both models demonstrated high consistency between the observed and anticipated values.

**Conclusion:**

The two nomogram models based on BMI and handgrip strength demonstrated good predictive ability for sarcopenia in male and female patients on PD. Subsequently, these may be used as convenient and inexpensive methods for the early detection and timely management of sarcopenia in patients on PD.

## 1 Introduction

Sarcopenia is characterized by an aging-related decline in muscle strength, skeletal muscle mass, and physical function (1). Patients receiving dialysis are vulnerable to sarcopenia owing to excess protein catabolism in the microinflammatory state, protein loss during dialysis, and insufficient protein intake (2). The prevalence of sarcopenia among patients on peritoneal dialysis (PD) in Asia and Europe ranges from 10.9 to 38.2% (3–5) and 4 to 17.7% (6, 7), respectively. In patients receiving dialysis, sarcopenia can increase the risk of falls and the incidence and mortality related to cardiovascular events, seriously affecting quality of life in patients (8, 9). Therefore, early screening for sarcopenia in patients on PD is crucial for nutritional management.

Sarcopenia is primarily diagnosed based on the criteria established by the European Working Group on Sarcopenia in Older People (EWGSOP) (10) and the Asian Working Group for Sarcopenia (AWGS) (1). Diagnostic testing comprises three aspects: muscular mass, muscle strength, and physical performance. Skeletal muscle mass can be estimated through magnetic resonance imaging (MRI), computed tomography (CT), dual-energy X-ray absorptiometry (DXA), and bioelectrical impedance analysis (BIA) (1). DXA is the most reliable technique for assessing muscular mass (11). BIA is commonly used to directly measure the skeletal muscle mass index (SMI), which is appendicular skeletal muscle mass modified based on the individual’s height in square meters (12). Muscle strength is generally assessed by determining handgrip strength (HGS) using handgrip dynamometry. The six-minute walk test (6MWT), five-repetition sit-to-stand (5-STS) test, short physical performance battery (SPPB), timed up-and-go (TUG) test, and other assessments are implemented to evaluate physical performance (10).

According to EWGSOP 2019, muscle strength is the primary indicator for diagnosing sarcopenia. Sarcopenia is characterized by a reduction in muscle mass and muscle strength. Physical function is an indicator for evaluating the severity of sarcopenia owing to its poor prognosis. According to AWGS2019, loss in physical function and muscle strength due to reduced muscle mass may adversely influence the prognosis. Sarcopenia may be diagnosed when the muscle mass, muscle strength, or physical function has declined. Furthermore, severe sarcopenia is diagnosed when muscle strength and physical function decline simultaneously. Therefore, decreased muscle mass is a necessary condition for diagnosing sarcopenia. Muscle strength or physical performance is one of the indicators used to diagnose sarcopenia and determine its severity.

The early diagnosis of sarcopenia in patients on PD is limited by the complication of applying the guidelines mentioned above. Furthermore, sarcopenia during dialysis differs from primary sarcopenia, as it can occur among young and middle-aged patients on dialysis, necessitating the identification of a predictive model for sarcopenia in individuals on PD.

In China, two prediction models are available for sarcopenia in patients on PD. Wu et al. (13) proposed that the total skeletal muscle and psoas muscle areas measured using computed tomography can be applied as variables to predict sarcopenia in patients on PD. The optimal thresholds for total skeletal muscle and psoas muscle index are 52.3 and 7.3 cm^2^/m^2^, respectively, for men and 36.3 and 6.1 cm^2^/m^2^, respectively, for women (13). Chen et al. (3) combined the phase angle with age to identify sarcopenia in patients on PD. In both sexes, the optimal thresholds for the phase angle and age were ≤ 5.3° and > 52 years, respectively. The optimal threshold for the phase angle was ≤5° and 5.3°, whereas the threshold for age was >54 years and 52 years for women and men, respectively.

The DXA method is the gold standard for assessing muscle mass but requires a DXA scanner. The DXA method is less convenient than the BIA method owing to the high cost of devices, unportability, dependence on highly trained operators, and the associated radiation exposure during radiology and computed tomography. Therefore, the EWGSOP guidelines do not recommend the use of DXA (10). Furthermore, the measurement of the phase angle requires a body composition analyzer, which is expensive and not routinely equipped in some countries or regions, leading to a lack of clinical attention for sarcopenia. Furthermore, its accuracy is affected by factors such as oedema, limb defects, and hemiplegia. However, these factors can bring sedentary behavior and physical inactivity in patients, making them more susceptible to sarcopenia (14, 15). Therefore, the false-negative rate for diagnosing sarcopenia using the BIA method may be high. Consequently, developing a simple and easily accessible method is crucial for sarcopenia screening.

A predictive model based on routine clinical examination data may be a simple and inexpensive method for screening sarcopenia. Body mass index (BMI) is a nutritional indicator that positively correlates with lean soft tissue (13) and appendicular lean mass index (16). Notably, a low BMI increases the likelihood of developing sarcopenia in patients on PD (17, 18) and could serve as a potential predictor of sarcopenia. The development of sarcopenia in patients on PD is closely associated with reduced protein synthesis and accelerated protein degradation, which is estimated using serum albumin, pre-albumin, ferroprotein, and hemoglobin levels (19).

HGS, which is used to evaluate upper limb muscle strength and is easy to measure, is a regularly used screening indicator for sarcopenia (20). The 5-STS test is performed to assess the power and flexibility of the muscles in the lower limbs. Compared with the 6MWT, SPPB, and other indicators of physical performance, HGS is relatively easy to measure and less time-consuming. Moreover, HGS can also be used to predict physical performance. Disturbances in serum calcium, serum phosphate, and parathyroid hormone levels can cause bone disorder (21). Abnormal serum potassium levels of chronic kidney disease result in muscle weakness, lassitude, and arrhythmia (22), which affect the patient’s physical function. Therefore, these routine follow-up indicators may be used as predictive indicators of sarcopenia.

The diagnosis of sarcopenia is limited by a shortage of diagnostic equipment in many clinics. A nomogram can simplify complex regression equations into an easy-to-read graph, which can be used to score the relative importance of every factor that impacts the outcome variable and subsequently calculate a total score to estimate the probability of certain result events (23, 24). Accordingly, it can serve as a basis for clinical decision-making. However, the prediction of sarcopenia in patients on PD using a nomogram remains unexplored. Therefore, this study aimed to develop and validate a convenient and practical technique for diagnosing sarcopenia in Chinese patients on PD based on routine follow-up examination data. Considering the obvious physiological differences in HGS between men and women, we constructed two sex-specific screening models. Binary sex categorization (male/female) was applied based on the sex designated at birth.

## 2 Materials and methods

### 2.1 Study design and participants

**Figure 1:**
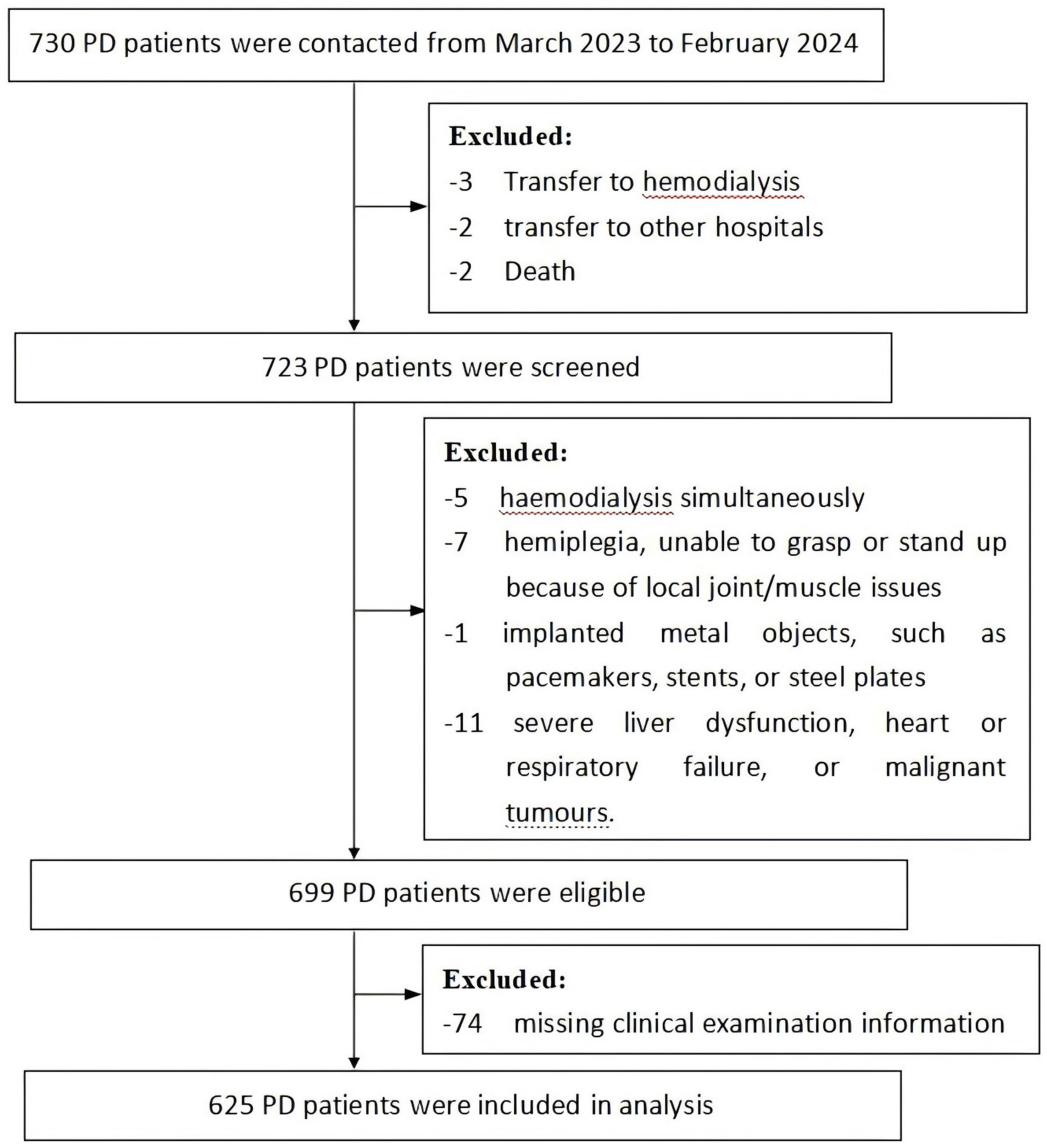
A flow diagram for the sample collection.

This cross-sectional study was conducted using a consecutive sample population of patients who had received PD and were followed-up regularly in the nephrology departments of two hospitals in Nanchang city, Jiangxi Province, China, from March 2023 to February 2024. The inclusion criteria were: ① age ≥ 18 years; ② regular peritoneal dialysis for ≥1 month; ③ the ability to communicate; and ④ the ability to undergo HGS measurement, the 5-STS test, and bioelectrical impedance measurement; ⑤ no protein supplements intake in the past 1 year. The exclusion criteria were: ① received hemodialysis simultaneously; ② hemiplegia, unable to grasp or stand up because of local joint/muscle issues; ③ presence of implanted metal objects, such as pacemakers, stents, or steel plates; ④ severe liver dysfunction, heart or respiratory failure, or malignant tumors. A flow diagram for the sample collection is shown in Figure 1.

The sample size was estimated according to the events per variable principle, implying that each predictor variable requires at least 10 outcome events (25). The sample size was 10 times the number of variables divided by the event occurrence rate. According to previous research, the prevalence of sarcopenia in Chinese patients on PD was approximately 23% (18), with seven predictive factors. Therefore, a sample size of 304 cases for each sex-specific model was estimated. Seventy-four records with incomplete follow-up data were excluded. The study enrolled a total of 625 participants. All the participants provided written informed consent. The study was approved by the Medical Ethics Committees of Nanchang University (approval code: 72264025) and the Medical Ethics Committees of Nanchang University First Affiliated Hospital (approval code: 2023CDYFYLK03-018).

### 2.2 Data collection

The data were collected by well-trained nurses who conducted onsite surveys and measurements based on a designed questionnaire, collecting data on demographics, dialysis adequacy indicators, laboratory examination indicators, HGS, SMI, and physical functional performance. These nurses were not involved in the research design and data analysis process.

For the diagnosis of sarcopenia, the AWGS2019 criteria were used by clinical doctors, which refers to low muscle mass accompanied by low muscle strength and/or physical performance (1). Low muscle mass refers to an SMI of <5.7 kg/m^2^ in women and < 7.0 kg/m^2^ in men. Low muscle strength refers to a HGS of <18 kg in women and < 28 kg in men. Low physical function refers to a 5-STS test result ≥12 s (1).

A body composition analyzer was used to assess SMI (InBody 270, Seoul, S. Korea). An electronic grip dynamometer (EH101; Camry Scales, Zhongshan, China) was used to evaluate HGS. The maximum reading was recorded among two successive measurements obtained using the dominant hand (26). The physical performance was evaluated using the 5-STS test. Participants crossed their hands over their chests and sat in a 40–43 cm chair. They were required to do five consecutive sit-downs and stand-ups once they heard the phrase ‘prepare, start’. Upon standing, their legs had to be straight. The shortest time, which was close to 0.01 s, would be recorded after the participants completed two tests. The 5-STS was terminated if the participant was unable to adhere to the requirements, adjusted their balance with their hands, or experienced shortness of breath (27).

Demographic data included age, sex, height, weight, and age at the time of dialysis. We performed statistical analysis for age as a continuous variable during the development of the predictor models, avoiding information loss. In addition, we categorized the age indicator into five additional groups: <35, 35–49, 50–59, 60–69, and >70 years, to further analyze the differences in the prevalence of sarcopenia among patients of different age groups. Weight (kg) was recorded using a body composition analyzer without dialysate, urine, heavy clothing, or shoes. The BMI was computed by dividing the weight (kg) by the square of the height (m^2^). Related indices of peritoneal dialysis, such as total urea clearance index, total creatinine clearance rate (Ccr), 4-h dialysate creatinine/plasma creatinine, and estimated glomerular filtration rate (eGFR), were calculated online using the Chinese blood purification registration system. The total urea clearance index is the sum of the clearance index of the residual kidney and that of the peritoneum. The total Ccr is the sum of the residual kidney Ccr and the peritoneal Ccr. Both indicators were used to assess the adequacy of dialysis. The type of peritoneal transport was assessed by a specialist nurse in accordance with the standard peritoneal equilibrium test procedures (28).

Venous blood was collected from the participants (fasting) on the follow-up day. The laboratory indices included albumin, hemoglobin, pre-albumin, ferritin, triglycerides, total cholesterol, high-density lipoprotein cholesterol (HDL-C), low-density lipoprotein cholesterol (LDL-C), calcium, phosphate, potassium, parathyroid hormone, and blood glucose. These indicators were used as potential predictors of sarcopenia in this study.

### 2.3 Statistical analysis

SPSS version 25.0 (IBM Corp., Armonk, NY, USA) and R version 4.0.1 (R Foundation for Statistical Computing, Vienna, Austria) were used for data analysis. Means and standard deviations were used to display data that were normally distributed. The interquartile ranges and medians were used to display data that were not normally distributed. Frequencies and percentages were used to represent categorical variables. The normally distributed data of two groups was contrasted using independent-sample *t*-tests. The Mann–Whitney *U* test was used to evaluate the non-normally distributed data in both groups, whereas the chi-square or Fisher’s exact test was applied to examine the categorical variables. A two-tailed *p* < 0.05 was considered statistically significant.

The preliminary factors associated with sarcopenia were selected through univariate analyses. Subsequently, multivariable stepwise logistic regression analyses of the selected factors (*p* < 0.1) were used to identify predictive factors. Nomogram models were constructed based on the multivariate logistic regression analysis. The model discriminative capacity was assessed using the area under the receiver operating characteristic curve (AUC). The AUC values were > 0.75, indicating good discriminatory ability of the models (29). The adequacy of the models for predicting sarcopenia in patients on PD was evaluated using the Hosmer–Lemeshow goodness-of-fit test, and the consistency between the predicted and observed values was assessed using calibration curves (30). Internal validation of the models was performed using 2000 bootstrap re-samplings.

## 3 Results

### 3.1 Prevalence of sarcopenia in patients on PD

Among the 625 participants receiving peritoneal dialysis, 87 were diagnosed with sarcopenia (13.92%) and 538 were not (86.08%).

### 3.2 Population characteristics

The average age of the participants was 48.30 ± 12.63 years. The characteristics of participants in the different sex groups are presented in Table 1. The characteristics of patients on PD in the different sex groups are presented in Table 2. The prevalence of sarcopenia was 35.3, 24.7, 11.3, 8.7, and 13.8% in patients aged >70, 60–90, 50–59, 35–49, and <35 years, respectively (Supplementary Table S1). The occurrence of sarcopenia exhibited a notable increase in both men and women aged 60 years compared to younger age groups.

**Table 1:** Baseline characteristics of the study population.

Variables		Participants (*n* = 625)	Man (*n* = 298)	Woman (*n* = 327)	*t/z/x^2^*	***p***
SMI (kg/m^2^)		6.97 ± 1.26	7.76 ± 0.07	6.32 ± 0.05	15.74^a^	< 0.01
HGS (kg)		26.91 ± 8.57	32.48 ± 0.45	21.84 ± 0.31	19.48^a^	< 0.01
5-STS [second, *M*(P_25_,P_75_)]		8.50 (6.76,10.23)	8.25 (6.53,10.12)	8.66 (6.85,10.43)	–1.37^b^	0.17
Age (years)		48.30 ± 12.63	48.11 ± 13.60	48.47 ± 11.70	–0.35^a^	0.73
BMI (kg/m^2^)		22.16 ± 3.20	22.37 ± 3.18	21.97 ± 3.21	1.56^a^	0.12
Dialysis vintage [months, *M*(P_25_,P_75_)]		28 (9,54)	20 (6,41)	33 (12,68)	–4.81^b^	< 0.01
daily urine output[ml, *M*(P_25_,P_75_)]		300 (0,800)	300 (0,800)	300 (0,700)	–1.53^b^	0.13
t-Kt/v [*M*(P_25_,P_75_)]		2.01 ± 0.74	1.55 ± 0.44	2.44 ± 0.70	−19.21^a^	< 0.01
t-Ccr [*M*(P_25_,P_75_)]		53.29 (44.78,68.03)	56.27 (46.65,77.67)	50.98 (43.44,63.91)	17.22^b^	< 0.01
eGFR [ml/min, *M*(P_25_,P_75_)]		1.36 (0,3.51)	1.47 (0,3.95)	1.15 (0,2.93)	1.74^b^	0.08
Types of peritoneal transport [*n*(%)]	High transport	40 (6.40)	25 (8.39)	15 (4.59)	–^c^	< 0.01
	High average	151 (24.16)	83 (27.85)	68 (20.80)		
	Mean	11 (1.76)	8 (2.68)	3 (0.92)		
	Low average	298 (47.68)	135 (45.30)	163 (49.84)		
	Low transport	125 (20.00)	47 (15.78)	78 (23.85)		
Alb (g/L)		37.53 ± 4.80	37.95 ± 4.92	37.15 ± 4.67	2.01^a^	0.04
Pre-Alb [mg/L, *M*(P_25_,P_75_)]		348.06 ± 81.03	359.53 ± 80.09	339.66 ± 80.64	3.11^a^	< 0.01
Ferritin [ug/L, *M*(P_25_,P_75_)]		152.0 (69.70,314.90)	184 (82.06,358.5)	125.5 (56.25,284.5)	3.69^b^	< 0.01
Hb (g/L)		113.74 ± 21.20	118.09 ± 22.59	109.77 ± 19.03	4.76^a^	< 0.01
TG [mmol/L, *M*(P_25_,P_75_)]		1.66 (1.17,2.37)	1.52 (1.05,2.16)	1.77 (1.27,2.64)	−4.05^b^	< 0.01
TC (mmol/L)		4.62 ± 1.11	4.34 ± 0.95	4.88 ± 1.18	−6.24^a^	< 0.01
HDL-C (mmol/L)		1.23 ± 0.38	1.18 ± 0.35	1.28 ± 0.39	−3.23^a^	< 0.01
LDL-C (mmol/L)		2.40 ± 0.81	2.24 ± 0.71	2.54 ± 0.86	−4.82^a^	< 0.01
BG (mmol/L)		6.08 ± 2.48	6.06 ± 2.02	6.09 ± 2.85	−0.19^a^	0.85
K (mmol/L)		3.99 ± 0.66	3.99 ± 0.68	3.99 ± 0.65	−0.07^a^	0.95
Ca (mmol/L)		2.28 ± 0.26	2.27 ± 0.29	2.29 ± 0.24	−0.84^a^	0.40
P (mmol/L)		1.60 ± 0.52	1.63 ± 0.58	1.58 ± 0.45	1.15^a^	0.25
PTH [pg/mL, *M*(P_25_,P_75_)]		338.0 (193.4,562.0)	322 (186.50,553.50)	370 (208,593)	−1.27^b^	0.21

a, *t*-value, independent-sample *t*-test; b, *z*-value, Mann–Whitney *U* test; c, Fisher’s exact test. Significant *p*-values (*p* < 0.05) are indicated in bold letter. SMI, skeletal muscle mass index; HGS, handgrip strength; 5-STS, five-repetition sit-to-stand test; BMI, body mass index; t-Kt/v, total urea clearance Index; t-Ccr, total creatinine clearance rate; eGFR, estimated glomerular filtration rate; Alb, albumin; Hb, hemoglobin; Pre-Alb, pre-albumin; ferritin, TG, triglyceride; TC, total cholesterol; HDL-C, high-density lipoprotein cholesterol; LDL-C, low-density lipoprotein cholesterol; Ca, serum calcium; P, serum phosphate; K, serum potassium; PTH, parathyroid hormone; BG, blood glucose.

**Table 2:** Characteristics of sarcopenia and non-sarcopenia in male and female PD patients.

Variables		Man (*n* = 298)		*t/z/x^2^*	*p*	Woman (n = 327)		*t/z/x^2^*	*p*
		Sarcopenia (*n* = 43)	Non-sarcopenia (*n* = 255)			Sarcopenia (*n* = 44)	Non-sarcopenia (*n* = 283)		
SMI (kg/m^2^)		6.18 ± 0.57	7.92 ± 1.10	−15.73^a^	<0.01	5.22 ± 0.42	6.49 ± 0.84	−15.90^a^	<0.01
HGS (kg)		24.38 ± 4.80	33.85 ± 7.33	−10.95^a^	<0.01	16.01 ± 3.19	22.75 ± 5.35	−11.70^a^	<0.01
5-STS [second, *M*(P_25_,P_75_)]		9.85 (8.04,13.04)	7.92 (6.44,9.91)	4.06^b^	<0.01	10.69 (8.78,12.98)	8.38 (6.76,10.00)	3.96^b^	<0.01
Age (years)		51.81 ± 16.53	47.49 ± 12.98	1.63^a^	0.11	51.84 ± 13.81	47.94 ± 11.27	1.78^a^	0.08
BMI (kg/m^2^)		19.41 ± 2.20	22.87 ± 3.05	−8.95^a^	<0.01	18.88 ± 1.76	22.45 ± 3.11	−11.01^a^	<0.01
Dialysis vintage [months, *M*(P_25_,P_75_)]		31 (9,63)	19 (6,40)	2.06^b^	<0.01	52.5 (20.5,85)	31 (11,63.5)	2.66^b^	<0.01
daily urine output [ml, *M*(P_25_,P_75_)]		100 (0,500)	400 (0,800)	−3.40^b^	<0.01	200 (0,500)	300 (0,735)	−1.94^b^	0.05
t-KT/V [*M*(P_25_,P_75_)]		1.53 ± 0.39	1.55 ± 0.45	0.30^a^	0.77	2.45 ± 0.60	2.44 ± 0.71	0.09^a^	0.94
t-Ccr [*M*(P_25_,P_75_)]		47.72 (42.85,58.21)	57.28 (48.86,76.59)	−3.59^b^	<0.01	48.17 (41.75,53.41)	51.70 (43.94,65.65)	−2.35^b^	<0.01
eGFR [ml/min, *M*(P_25_,P_75_)]		0.57 (0,2.86)	1.55 (0,4.41)	−2.31^b^	0.02	0.62 (0,1.84)	1.32 (0,3.22)	−1.98^b^	<0.05
Types of peritoneal transport [*n*(%)]	High transport	2 (4.65)	23 (9.02)	–^c^	0.75	2 (4.54)	13 (4.59)	–^c^	0.81
	High average	12 (27.91)	71 (27.84)			12 (27.27)	56 (19.79)		
	Mean	2 (4.65)	6 (2.35)			0 (0)	3 (1.06)		
	Low average	21 (48.84)	114 (44.71)			20 (45.46)	143 (50.53)		
	Low transport	6 (13.95)	41 (16.08)			10 (22.73)	68 (24.03)		
Alb (g/L)		37.23 ± 5.79	38.07 ± 4.76	−1.03^a^	0.31	36.99 ± 4.69	37.18 ± 4.67	−0.24^a^	0.81
Pre-Alb [mg/L, *M*(P_25_,P_75_)]		356.75 ± 86.54	359.08 ± 79.18	−0.18^a^	0.86	331.23 ± 110.55	338.66 ± 75.31	−0.19^a^	0.85
Ferritin [ug/L, *M*(P_25_,P_75_)]		169 (80.05,253.65)	190 (81.51,361)	0.87^b^	0.38	136 (44.8,371.95)	125 (59.15,274.5)	0.24^b^	0.81
Hb (g/L)		124.12 ± 21.62	117.08 ± 22.63	1.90^a^	0.06	110.91 ± 21.92	109.61 ± 18.58	0.42^a^	0.67
TG [mmol/L, *M*(P_25_,P_75_)]		1.67 (1.11,2.03)	1.5 (1.03,2.17)	4.60^b^	0.84	1.94 (1.40,2.33)	1.76 (1.27,2.66)	0.41^b^	0.69
TC (mmol/L)		4.46 ± 1.02	4.32 ± 0.94	0.94^a^	0.35	4.94 ± 1.40	4.87 ± 1.15	0.04^a^	0.69
HDL-C (mmol/L)		1.26 ± 0.53	1.17 ± 0.32	1.09^a^	0.28	1.29 ± 0.27	1.28 ± 0.40	0.22^a^	0.83
LDL-C (mmol/L)		2.32 ± 0.78	2.23 ± 0.70	0.81^a^	0.42	2.65 ± 0.93	2.53 ± 0.85	0.91^a^	0.37
BG (mmol/L)		6.13 ± 2.12	6.04 ± 1.99	0.26^a^	0.80	6.55 ± 3.96	6.02 ± 2.64	1.14^a^	0.26
K (mmol/L)		3.64 ± 0.67	4.04 ± 0.66	−3.70^a^	< 0.01	3.82 ± 0.58	4.02 ± 0.65	−1.91^a^	0.06
Ca (mmol/L)		2.26 ± 0.20	2.27 ± 0.30	−0.18^a^	0.86	2.25 ± 0.31	2.29 ± 0.23	−1.21^a^	0.23
P (mmol/L)		1.55 ± 0.67	1.64 ± 0.60	−0.90^a^	0.37	1.62 ± 0.43	1.57 ± 0.45	−0.58^a^	0.56
PTH [pg/mL, *M*(P_25_,P_75_)]		382 (194,769.5)	320 (187.5,490.5)	1.56^b^	0.12	384 (192,663.5)	358 (214.1,562)	0.47^b^	0.64

a, *t*-value, independent-sample *t*-test; b, *z*-value, Mann–Whitney U test; c, Fisher’s exact test. SMI, skeletal muscle mass index; HGS, handgrip strength; 5-STS, five-repetition sit-to-stand test; BMI, body mass index; t-Kt/v, total urea clearance Index; t-Ccr, total creatinine clearance rate; eGFR, estimated glomerular filtration rate; Alb, albumin; Hb, hemoglobin; Pre-Alb, pre-albumin; ferritin, TG, triglyceride; TC, total cholesterol; HDL-C, high-density lipoprotein; LDL-C, low-density lipoprotein; Ca, serum calcium; P, serum phosphate; K, serum potassium; PTH, parathyroid hormone; BG, blood glucose. Significant *p*-values (*p* < 0.05) are indicated in bold letters.

Male patients had significantly higher SMI, HGS, total Ccr, and albumin, pre-albumin, ferritin, and hemoglobin levels than female patients (all *p* < 0.05). The dialysis age of female patients was higher than that of male patients. Additionally, female patients had greater total urea clearance index, triglyceride, total cholesterol, and HDL-C, and LDL-C levels than male patients (all *p* < 0.05). Furthermore, differences in the distribution of peritoneal transport types were observed between two groups (Table 1). These findings indicated that sex-based classification was more appropriate for the development of a PD sarcopenia prediction model.

### 3.3 Univariate analysis of sex-specific data

The sarcopenia and non-sarcopenia groups were established for the different sexes (Table 2). The study findings revealed that the prevalence of sarcopenia among men and women receiving PD was 14.43 and 13.46%, respectively. The chi-square test exhibited no statistically significant distinction between the two groups (*x*^2^ = 0.123, *p* = 0.725). In men and women, individuals with sarcopenia had lower SMI, HGS, BMI, residual urine output, total Ccr, and eGFR than those without sarcopenia. The duration of the 5-STS test was longer, and the age of dialysis was higher in the sarcopenia group than in the non-sarcopenia group (all *p* < 0.05). Furthermore, blood potassium levels were significantly lower in men among the sarcopenia group than in the non-sarcopenia group (*p* < 0.01).

### 3.4 Multiple regression analysis of sex-specific data

A stepwise multiple regression analysis was performed using indicators with *p* < 0.1 from the univariate analyses as independent variables and sarcopenia as the dependent variable (non-sarcopenia = 0, sarcopenia = 1). In male and female patients who received peritoneal dialysis, the findings suggested that HGS and BMI were independent risk factors for sarcopenia (Table 3).

**Table 3:** Multivariate analysis.

Gender	Variables	*β*	SE	OR (95CI%)	*p*
Men	HGS	−0.25	0.04	0.78 (0.72–0.85)	< 0.01
	BMI	−0.50	0.10	0.61 (0.50–0.74)	< 0.01
Women	HGS	−0.44	0.07	0.64 (0.56–0.74)	< 0.01
	BMI	−0.62	0.11	0.54 (0.43–0.67)	< 0.01

HGS, handgrip strength; BMI, body mass index; SE, standard error; OR, odds ratio; CI, confidence interval.

### 3.5 Construction of the nomogram models

**Figure 2:**
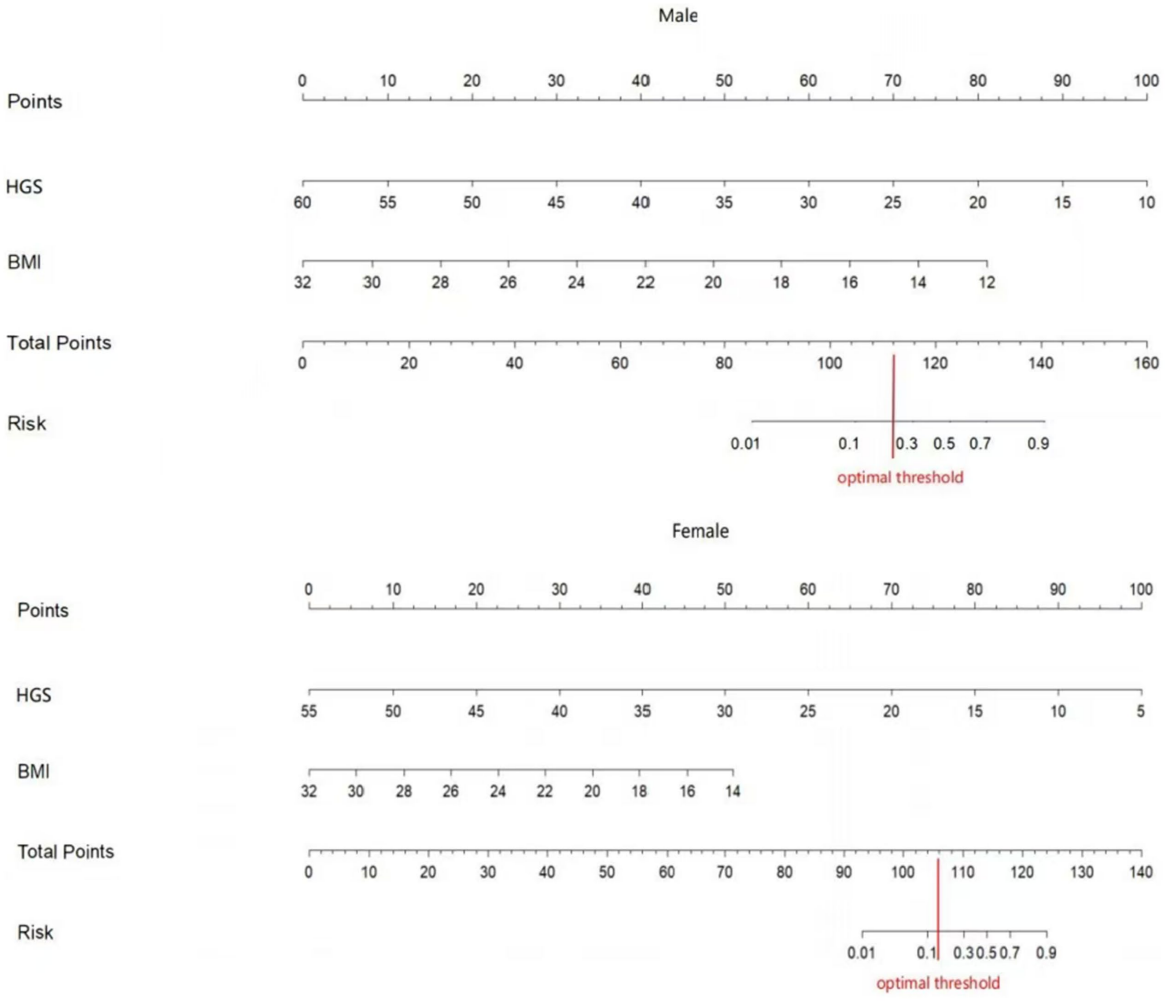
The nomogram prediction models for sarcopenia in male and female PD patients.

**Figure 3:**
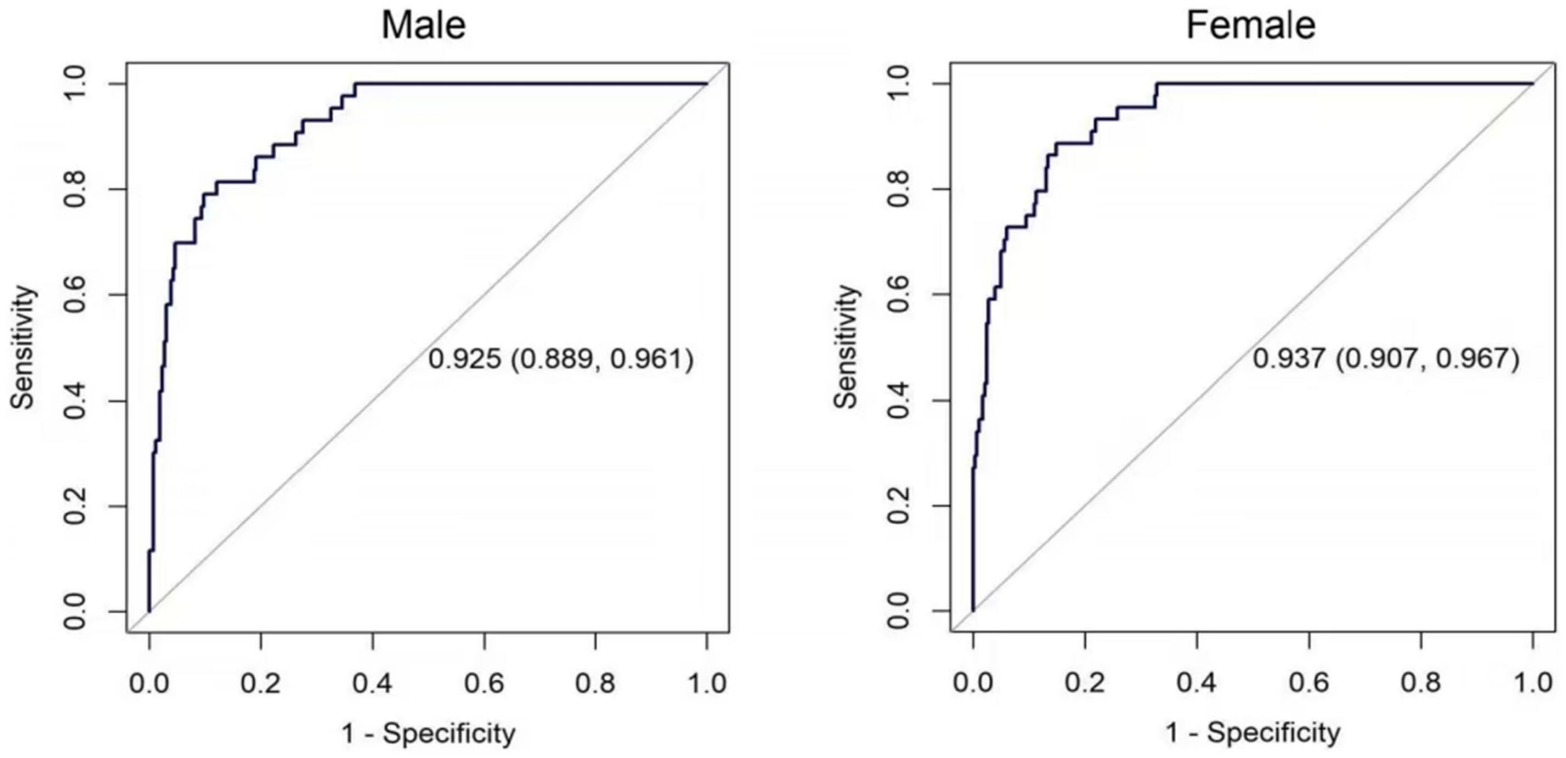
ROCS of the prediction models for sarcopenia in male and female PD patients.

**Figure 4:**
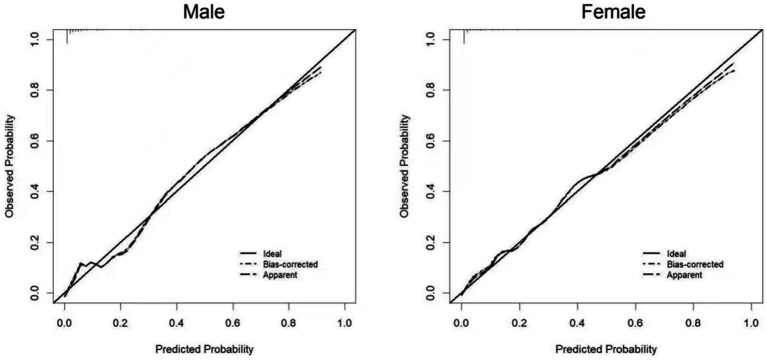
Calibration curves of the prediction models for sarcopenia in male and female PD patients.

Two nomograms were constructed using the results from the multivariate analysis. The two nomogram models of sarcopenia in men and women are shown in Figure 2. The AUC of the male predictive model was 0.925 [95% confidence interval (CI): 0.889–0.961], with an accuracy of 88.59%, sensitivity of 79.07%, and specificity of 90.20% (Supplementary Table S2). The optimal threshold of the male predictive model was 0.242, corresponding to approximately 110 HGS + BMI points (Figure 2). In the internal validation, the AUC of the model was 0.924 (95% CI: 0.888–0.959). The AUC of the female predictive model was 0.937 (95% CI: 0.907–0.967), with an accuracy of 85.63%, sensitivity of 88.64%, and specificity of 85.16% (Supplementary Table S3). In the internal validation, the AUC of the model was 0.936 (95% CI: 0.906–0.966) (Figure 3). The optimal threshold of the female model was 0.149, corresponding to approximately 106 HGS + BMI points. The calibration curves of the two models revealed good agreement between the predicted and observed values. The Hosmer–Lemeshow test exhibited *x^2^* = 5.210 (*p* = 0.735) for the male prediction model and *x*^2^ = 2.057 (*p* = 0.979) for the female prediction model, indicating remarkable calibration of both models (Figure 4). For example, a female patient on PD had HGS of 16 kg and BMI of 18 kg/m^2^, scoring 118 total points, which exceeded the threshold of 106, as the HGS score was 78 and the BMI score was 40. Therefore, the female patient would be diagnosed with sarcopenia (Figure 2).

## 4 Discussion

Sarcopenia is a common condition observed in patients on PD. It serves as an indicator for cardiovascular events and mortality in these patients (31). The early identification of high-risk patients guides optimal clinical decisions by professionals for their patients, which is of great clinical significance. Certain models are available for the prediction of sarcopenia in patients on PD (13, 32–34). Although these prediction methods are simple and effective, they require expensive equipment, thus necessitating the exploration of a cost-effective and simple screening method for sarcopenia in patients on PD.

### 4.1 Principal findings

We selected indicators that may be related to the diagnostic components of AWGS 2019 as potential predictive variables from routine follow-up examinations performed in the nephrology departments of two hospitals in a city in south-eastern China. Owing to the significant difference in HGS between men and women, we constructed two independent nomogram models for sarcopenia for male and female patients. The results showed that HGS and BMI were key predictive indicators of sarcopenia in men and women. The models exhibited good discriminability and calibration. To our knowledge, these are the first prediction models that use a nomogram and two easily obtainable indicators (HGS and BMI) to predict sarcopenia in patients on PD.

### 4.2 Prevalence of sarcopenia among patients on PD

According to the findings of our cross-sectional survey, the prevalence of sarcopenia in patients on PD was 13.92%, which is lower than the previous estimate. Considering the different medical conditions in different regions, the sample size was determined by referencing a Chinese study from Ningbo city, whose economic and medical levels were comparable to those of our city. Notably, the data for that study were collected during 2017. Advancements in medical technology and enhanced dietary understanding among patients might contribute to a reduction in the prevalence of illness. Furthermore, the diagnostic criteria for sarcopenia were based on the AWGS 2014, and the average age of patients was 56.1 years, which was higher than that of our participants. However, the prevalence of sarcopenia in patients on PD in our study is consistent with the findings of an additional Chinese study from a developed city, where the average patient age was 55.3 years (35). Therefore, we compared our findings with those of other Chinese studies (3, 31) in which the diagnostic criteria for sarcopenia were consistent with those used in our study. The average age of the patients in the studies conducted by Wu et al. (31) and Chen et al. (3) was 54.25 and 51.31 years, and the prevalence of sarcopenia was 31.43 and 29.70%, respectively. This indicates that the prevalence of sarcopenia varies substantially among regions of the same country. This may be attributed to the differences in patient characteristics, diagnostic criteria, economic conditions, and medical and health resources in the different regions included in each study.

### 4.3 Predictive factors for sarcopenia among patients on PD

#### 4.3.1 HGS and 5-STS

HGS serves as a reliable measure of the strength in the upper limbs and is convenient and easy to perform. The strength of the muscles in the lower limbs can be most accurately measured by assessing the flexion and extension strength of the knee joint, which necessitates the use of an isokinetic muscular strength tester (10). However, the device is expensive and complex. Therefore, the 5-STS test instead of measuring lower-limb strength was used in this study owing to its relative ease of operation. As shown in the nomogram models, HGS is a predictive indicator of sarcopenia in male and female patients on PD and had the greatest impact on the occurrence of sarcopenia. Therefore, HGS can be considered as a measure of muscle strength in patients on PD with sarcopenia.

HGS is a globally recognized indicator of muscular strength. Wu et al. (31) extracted 12 core indicators for predicting sarcopenia based on machine learning models, including HGS and BMI. HGS was significantly correlated with skeletal muscle mass among patients on PD with sarcopenia (36). It is a reliable indicator for predicting the prognosis of PD sarcopenia. Xu et al. (37) used HGS, with thresholds of <24.5 kg for men and <14.0 kg for women, as well as lean mass index, with thresholds of <16.7 kg/m^2^ for men and <13.8 kg/m^2^ for women, to anticipate the risk of all-cause mortality in patients on PD with sarcopenia. The predictive ability was more optimized than the recommended HGS value from the AWGS 2019. The HGS of patients with sarcopenia was positively correlated with the cross-sectional area and thickness of the gastrocnemius muscle (38) and linearly correlated with the strength of all lower-limb muscle groups (39). Compared with the upper limbs, the lower limbs exhibit a more rapid reduction in muscle strength (40). Therefore, a decrease in HGS indicates a prior loss in muscle strength in the lower limbs.

HGS is also linked to the physical performance of patients on PD. Umakanthan et al. (36) reported a direct relationship between HGS and the outcomes of the TUG test in patients on dialysis with sarcopenia. Despite the 5-STS test being an indicator for predicting physical performance and its association with sarcopenia in univariate analysis, it was excluded from the regression equation in multivariate analysis. A clinical trial showed that drug intervention can change muscle mass but have no effect on muscle function. Physical function performance is changed when muscle mass improves to a certain degree (41). This suggests that HGS could serve as a more sensitive indicator of muscle strength and functional performance in patients on PD with sarcopenia. Therefore, the 5-STS test indicator was not included in the sarcopenia prediction model.

Overhydration, presented as facial or lower limb oedema, significant weight gain, hypertension, dyspnoea, chest constriction, and more symptoms, is a common concern in patients on PD. Overhydration is prone to hypertension and characterized by dizziness and headaches. It limits the patient’s willingness and ability of activities (42). Overhydration can cause severe consequences, including heart and respiratory failure, restricting the patient’s mobility (43, 44). Conversely, patients experiencing dehydration feel fatigue, muscle convulsions, and nausea, which also adversely impact their physical performance. The participants of this study could perform grip strength and 5-STS tests, but those with severe heart failure or respiratory failure were excluded. Therefore, this may underestimate the prevalence of sarcopenia in patients on PD.

#### 4.3.2 BMI

BMI and albumin, ferritin, pre-albumin, and hemoglobin levels are widely used to assess patients’ nutritional condition during PD. We found no significant differences in albumin, ferritin, pre-albumin, or hemoglobin levels between individuals with and without sarcopenia. Only BMI had a predictive value, which is consistent with the results of two Chinese studies (15, 16). Patients on PD are prone to a low BMI, low muscle mass, and even malnutrition owing to a decreased appetite, dietary control, and nutrient absorption disorders. Being underweight is substantially related to a higher risk of sarcopenia (45). Furthermore, the higher the BMI, the lower the risk of sarcopenia (46). However, Wang et al. (47) discovered a correlation between BMI and sarcopenia in older adults with chronic conditions that follows a U-shaped pattern. Specifically, the incidence of sarcopenia increased when the BMI was >27.1 kg/m^2^. This might be attributed to skeletal muscle fat infiltration or muscle steatosis (48, 49).

In this study, we observed no differences in triglyceride, total cholesterol, HDL-C, and LDL-C between patients on PD with and without sarcopenia, which is similar to the findings of other studies (15, 16). Regardless of the use of replacement renal therapy, sarcopenia in patients with chronic kidney disease is linked to a low BMI (50). Moreover, sarcopenic obesity is rare in patients with a BMI of >30 kg/m^2^, and none in patients on hemodialysis (50). Notably, no patients with sarcopenia exhibited a BMI of >30 kg/m^2^ among the 625 patients on PD in this study. Therefore, the occurrence of sarcopenia in patients on PD is linearly related to a decreased BMI. Future studies should investigate the relationship between BMI and indicators such as blood lipids in patients on PD with sarcopenic obesity.

#### 4.3.3 Age

In this study, the predictive model did not include the age factor. The results demonstrated that individuals aged ≥60 years receiving PD were at a high risk of developing sarcopenia, regardless of their sex (Supplementary Table S1). Primary sarcopenia is closely associated with age (51). Although sarcopenia in patients receiving dialysis is secondary, primary sarcopenia cannot be ruled out in certain older patients. Chen et al. (3) observed that the occurrence of sarcopenia in patients on PD increases with age, and the prevalence of sarcopenia changed with age in both men and women. This could be attributed to the participants’ average age of 48 years, with a comparatively small number of individuals aged >60 years. Therefore, patients aged >60 years should be particularly vigilant about the occurrence of sarcopenia.

## 5 Clinical relevance and limitations

The sarcopenia prediction model for male and female patients on PD developed in this study could aid in the clinical diagnosis of sarcopenia. Sarcopenia may be diagnosed when the total score of BMI and HGS exceeds 110 in men and 106 in women. Our nomogram models are capable of predicting the present risk probability of sarcopenia in patients on PD. For patients at high risk of sarcopenia, intensive clinical surveillance, early exercise, and nutritional intervention may be adopted to mitigate poor effects. HGS testing is simple and more suitable for widespread application in the primary care hospitals. The samples in this study were sourced from two hospitals in a single city in China, and the performance evaluation of the prediction model involved the self-sampling strategy, which may affect the external validity of the results. Therefore, caution is advised when extrapolating these findings to patients from other nations or regions. Additionally, further verification of the models is required. Researchers in future studies should increase the sample size and perform a nationwide multicenter study to investigate the factors associated with PD sarcopenia in various age groups. Moreover, the cross-sectional design employed in this study reduces the ability to infer causality between variables. Researchers in future studies should explore the causal relationship among variables using the longitudinal study design.

## 6 Conclusion

The two nomogram models based on BMI and HGS developed in this study can predict the risk of sarcopenia in Chinese male and female patients on PD. The nomogram models demonstrated good discrimination and calibration. The indicators of BMI and HGS are conveniently measurable, inexpensive, and easy to apply in grassroots hospitals. To our knowledge, our sample size was sufficiently large compared to those used in other studies, and we developed the first simple model for predicting sarcopenia based on regular follow-up data of patients on PD. These models facilitate the rapid identification of sarcopenia and are crucial in guiding clinical decisions to prevent and reverse sarcopenia in patients on PD.

## Data Availability

The original contributions presented in the study are included in the article/Supplementary material, further inquiries can be directed to the corresponding author.

## References

[1] Chen LK Woo J Assantachai P Auyeung TW Chou MY Iijima K. Asian working Group for Sarcopenia: 2019 consensus update on sarcopenia diagnosis and treatment. J Am Med Dir Assoc. (2020) 21:300–307.e2. doi: 10.1016/j.jamda.2019.12.012, PMID: 32033882

[2] Cruz-Jentoft AJ Baeyens JP Bauer JM Boirie Y Cederholm T Landi F. Sarcopenia: European consensus on definition and diagnosis: report of the European working group on sarcopenia in older people. Age Ageing. (2010) 39:412–23. doi: 10.1093/ageing/afq034, PMID: 20392703 PMC2886201

[3] Chen Y Wu J Ran L Yu D Chen X Liu M. The combination of phase angle and age has a good diagnostic value for sarcopenia in continuous ambulatory peritoneal dialysis patients. Front Nutr. (2022) 9:1036796. doi: 10.3389/fnut.2022.1036796, PMID: 36458164 PMC9705784

[4] Kamijo Y Kanda E Ishibashi Y Yoshida M. Sarcopenia and frailty in PD: impact on mortality, malnutrition, and inflammation. Perit Dial Int. (2018) 38:447–54. doi: 10.3747/pdi.2017.00271 30065064

[5] Lin YL Wang CH Tsai JP Chen CT Chen YH Hung SC. A comparison of SARC-F, calf circumference, and their combination for sarcopenia screening among patients undergoing peritoneal dialysis. Nutrients. (2022) 14:923. doi: 10.3390/nu14050923, PMID: 35267898 PMC8912378

[6] Silva MZC Vogt BP Reis NSC Caramori JCT. Correction: update of the European consensus on sarcopenia: what has changed in diagnosis and prevalence in peritoneal dialysis? Eur J Clin Nutr. (2020) 74:357–8. doi: 10.1038/s41430-019-0538-2, PMID: 31831841

[7] Davenport A. Comparison of frailty, sarcopenia and protein energy wasting in a contemporary peritoneal dialysis cohort. Perit Dial Int. (2022) 42:571–7. doi: 10.1177/08968608221077462 35289199

[8] Chen LK Liu LK Woo J Assantachai P Auyeung TW Bahyah KS. Sarcopenia in Asia: consensus report of the Asian working Group for Sarcopenia. J Am Med Dir Assoc. (2014) 15:95–101. doi: 10.1016/j.jamda.2013.11.025, PMID: 24461239

[9] Baltacı MA Atmis V Metin Y Aktar M Eren SA Sengul S. Sarcopenia and cardiovascular risk indices: its impact on cardiovascular events and mortality in dialysis patients. Semin Dial. (2023) 36:221–30. doi: 10.1111/sdi.13106, PMID: 35706153

[10] Cruz-Jentoft AJ Bahat G Bauer J Boirie Y Bruyère O Cederholm T. Writing Group for the European Working Group on sarcopenia in older people 2 (EWGSOP2), and the extended group for EWGSOP2. Sarcopenia: revised European consensus on definition and diagnosis. Age Ageing. (2019) 48:16–31. doi: 10.1093/ageing/afy169, PMID: 30312372 PMC6322506

[11] Buckinx F Landi F Cesari M Fielding RA Visser M Engelke K. Pitfalls in the measurement of muscle mass: a need for a reference standard. J Cachexia Sarcopenia Muscle. (2018) 9:269–78. doi: 10.1002/jcsm.12268, PMID: 29349935 PMC5879987

[12] Kim KM Jang HC Lim S. Differences among skeletal muscle mass indices derived from height-, weight-, and body mass index-adjusted models in assessing sarcopenia. Korean J Intern Med. (2016) 31:643–50. doi: 10.3904/kjim.2016.015, PMID: 27334763 PMC4939509

[13] Wu CH Chao CT Liang PC Shih TTF Huang JW. Computed tomography-based sarcopenia in patients receiving peritoneal dialysis: correlation with lean soft tissue and survival. J Formos Med Assoc. (2022) 121:500–9. doi: 10.1016/j.jfma.2021.06.026, PMID: 34274192

[14] Kerr NR Booth FW. Contributions of physical inactivity and sedentary behavior to metabolic and endocrine diseases. Trends Endocrinol Metab. (2022) 33:817–27. doi: 10.1016/j.tem.2022.09.002, PMID: 36283907

[15] Zhang Y Liu X Ma Y Li X. Physical activity, sedentary behavior, fruit and vegetable consumption, and sarcopenia in older Chinese adults: a cross-sectional study. Nutrients. (2023) 15:3417. doi: 10.3390/nu15153417, PMID: 37571354 PMC10420903

[16] Do JY Kim AY Kang SH. Clinical usefulness of neck circumference for predicting sarcopenia in patients undergoing peritoneal dialysis. Nutr Clin Pract. (2022) 37:1366–75. doi: 10.1002/ncp.10886, PMID: 35780314

[17] Sun X Li X Wang J Jia L Ma Y Li F. Incidence and related factors of myopenia in middle-aged and elderly peritoneal dialysis patients. J Clin Nephrol. (2023) 23:727–31. doi: 10.3969/j.issn.1671-2390.2023.09.004

[18] Zhu B Zhou F Ye H Xue C Lu M Luo Q. Risk factors of sarcopenia in patients receiving maintenance peritoneal dialysis. Chin J Gen Pract. (2020) 19:913–7. doi: 10.3760/cma.j.cn114798-20191231-00923

[19] Massini G Caldiroli L Molinari P Carminati FMI Castellano G Vettoretti S. Nutritional strategies to prevent muscle loss and sarcopenia in chronic kidney disease: what Do we currently know? Nutrients. (2023) 15:3107. doi: 10.3390/nu15143107, PMID: 37513525 PMC10384728

[20] Dunn JA Koch-Borner S Johanson ME Wangdell J. Toward consensus in assessing upper limb muscle strength and pinch and grip strength in people with tetraplegia having upper limb reconstructions. Top Spinal Cord Inj Rehabil. (2021) 27:70–82. doi: 10.46292/sci20-00012, PMID: 34456548 PMC8370705

[21] Ketteler M Block GA Evenepoel P Fukagawa M Herzog CA McCann L. Executive summary of the 2017 KDIGO chronic kidney disease-mineral and bone disorder (CKD-MBD) guideline update: what's changed and why it matters. Kidney Int. (2017) 92:26–36. https://10.1016/j.kint.2017.04.006. doi: 10.1016/j.kint.2017.04.006, PMID: 28646995

[22] Yamada S Inaba M. Potassium metabolism and Management in Patients with CKD. Nutrients. (2021) 13:1751. doi: 10.3390/nu13061751, PMID: 34063969 PMC8224083

[23] Bonnett LJ Snell KIE Collins GS Riley RD. Guide to presenting clinical prediction models for use in clinical settings. BMJ. (2019) 365:l737. doi: 10.1136/bmj.l737 30995987

[24] Park SY. Nomogram: an analogue tool to deliver digital knowledge. J Thorac Cardiovasc Surg. (2018) 155:1793. doi: 10.1016/j.jtcvs.2017.12.107, PMID: 29370910

[25] Riley RD Snell KI Ensor J Burke DL Harrell FE Jr Moons KG. Minimum sample size for developing a multivariable prediction model: PART II-binary and time-to-event outcomes. Stat Med. (2019) 38:1276–96. doi: 10.1002/sim.7992, PMID: 30357870 PMC6519266

[26] Lee SC Wu LC Chiang SL Lu LH Chen CY Lin CH. Validating the capability for measuring age-related changes in grip-force strength using a digital hand-held dynamometer in healthy young and elderly adults. Biomed Res Int. (2020) 2020:6936879–9. doi: 10.1155/2020/6936879, PMID: 32382565 PMC7191369

[27] Jones SE Kon SS Canavan JL Patel MS Clark AL Nolan CM. The five-repetition sit-to-stand test as a functional outcome measure in COPD. Thorax. (2013) 68:1015–20. doi: 10.1136/thoraxjnl-2013-203576, PMID: 23783372

[28] Twardowski ZJ Nolph KD Khanna R Prowant BF Ryan LR Moore HL. Peritoneal equilibration test. Perit Dial Bull. (1987) 7:138–48. doi: 10.1111/j.1525-139x.1990.tb00029.x

[29] Mei Z Chen J Chen P Luo S Jin L Zhou L. A nomogram to predict hyperkalemia in patients with hemodialysis: a retrospective cohort study. BMC Nephrol. (2022) 23:351. doi: 10.1186/s12882-022-02976-4, PMID: 36319967 PMC9628065

[30] Mo YH Su YD Dong X Zhong J Yang C Deng WY. Development and validation of a nomogram for predicting sarcopenia in community-dwelling older adults. J Am Med Dir Assoc. (2022) 23:715–721.e5. doi: 10.1016/j.jamda.2021.11.023, PMID: 34932988

[31] Wathanavasin W Banjongjit A Avihingsanon Y Praditpornsilpa K Tungsanga K Eiam-Ong S. Prevalence of sarcopenia and its impact on cardiovascular events and mortality among dialysis patients: a systematic review and meta-analysis. Nutrients. (2022) 14:4077. doi: 10.3390/nu14194077, PMID: 36235729 PMC9572026

[32] Wu J Guan J Lin S Wu X Ding M Ren Z. Prediction of sarcopenia among peritoneal dialysis patients using a combination of irisin and phase angle. Nephrol Ther. (2023) 19:66–75. doi: 10.1684/ndt.2023.7, PMID: 36880103

[33] Wu J Lin S Guan J Wu X Ding M Shen S. Prediction of the sarcopenia in peritoneal dialysis using simple clinical information: a machine learning-based model. Semin Dial. (2023) 36:390–8. doi: 10.1111/sdi.13131, PMID: 36890621

[34] Do JY Kim AY Kang SH. Association between phase angle and sarcopenia in patients undergoing peritoneal dialysis. Front Nutr. (2021) 8:742081. doi: 10.3389/fnut.2021.742081, PMID: 34631771 PMC8497817

[35] Shen YW Su XY Liu M Yu Z Yan H Ma D. Prevalence and risk factors of sarcopenia in peritoneal dialysis patients. Chin J Nephrol. (2019) 35:268–74. doi: 10.3760/cma.j.issn.1001-7097.2019.04.005

[36] Umakanthan M Li JW Sud K Duque G Guilfoyle D Cho K. Prevalence and factors associated with sarcopenia in patients on maintenance dialysis in Australia—a single Centre, cross-sectional study. Nutrients. (2021) 13:3284. doi: 10.3390/nu13093284, PMID: 34579163 PMC8469859

[37] Xu X Yang Z Ma T Li Z Chen Y Zheng Y. The cut-off values of handgrip strength and lean mass index for sarcopenia among patients on peritoneal dialysis. Nutr Metab (Lond). (2020) 17:84. doi: 10.1186/s12986-020-00506-3, PMID: 33062032 PMC7542899

[38] Wei W Xie C Cao R Que Y Zhong X Chen Z. Ultrasound assessment of the gastrocnemius muscle as a potential tool for identifying sarcopenia in patients with type 2 diabetes. Diabetes Metab Syndr Obes. (2023) 16:3435–44. doi: 10.2147/DMSO.S435517, PMID: 37929058 PMC10624255

[39] Strandkvist V Larsson A Pauelsen M Nyberg L Vikman I Lindberg A. Hand grip strength is strongly associated with lower limb strength but only weakly with postural control in community-dwelling older adults. Arch Gerontol Geriatr. (2021) 94:104345. doi: 10.1016/j.archger.2021.104345 33497911

[40] Liu J Ding Q Zhou B Liu X Liu J Liu Y. Geriatric branch of Chinese Medical Association, editorial Committee of Chinese Journal of geriatric medicine. Expert consensus on diagnosis and treatment of sarcopyosis in China. *Chinese*. J Geriatrics. (2021) 40:943–52. doi: 10.3760/cma.j.issn.0254-9026.2021.08.001

[41] Woodhouse L Gandhi R Warden S Poiraudeau S Myers SL Benson CT. A Phase 2 randomizedstudy investigating the efhcacy and safety of myostatin antibody LY2495655 versus placebo in patients undergoing elective totalhip arthroplasty.J Frailty Aging. (2016) 5, 1–9. doi: 10.14283/jfa.2016.81 26980371

[42] Skow LF Sharrett AR Gottesman RF Coresh J Deal JA Palta P. Mid-life vascular risk and rate of physical function decline among older adults: the atherosclerosis risk in communities (ARIC) study. J Gerontol A Biol Sci Med Sci. (2024) 79:glad210. doi: 10.1093/gerona/glad210, PMID: 37659100 PMC10809050

[43] Yakut OH Bozdemir OC Dural M Yalvac HE Al A Murat S. The 6-minute walk test and fall risk in patients with heart failure: a cross-sectional study. Heart Lung. (2024) 64:80–5. doi: 10.1016/j.hrtlng.2023.11.012, PMID: 38065041

[44] Herridge MS Cheung AM Tansey CM Matte-Martyn A Diaz-Granados N al-Saidi F. Canadian critical care trials group. One-year outcomes in survivors of the acute respiratory distress syndrome. N Engl J Med. (2003) 348:683–93. doi: 10.1056/NEJMoa022450 12594312

[45] Curtis M Swan L Fox R Warters A O'Sullivan M. Associations between body mass index and probable sarcopenia in community-dwelling older adults. Nutrients. (2023) 15:1505. doi: 10.3390/nu15061505, PMID: 36986233 PMC10059806

[46] Merchant RA Seetharaman S Au L Wong MWK Wong BLL Tan LF. Relationship of fat mass index and fat free mass index with body mass index and association with function, cognition and sarcopenia in pre-frail older adults. Front Endocrinol. (2021) 12:765415. doi: 10.3389/fendo.2021.765415, PMID: 35002957 PMC8741276

[47] Wang N Wei Y Liu J Wang J. Risk factors for sarcopenia in elderly hospitalized patients with chronic diseases. Chin Gen Pract. (2020) 23:611–6. doi: 10.12114/j.issn.1007-9572.2020.00.006

[48] Al-Saedi A Debruin DA Hayes A Hamrick M. Lipid metabolism in sarcopenia. Bone. (2022) 164:116539. doi: 10.1016/j.bone.2022.116539 36007811

[49] Lin Y Zhong S Sun Z. Association between serum triglyceride to high-density lipoprotein cholesterol ratio and sarcopenia among elderly patients with diabetes: a secondary data analysis of the China health and retirement longitudinal study. BMJ Open. (2023) 13:e075311. doi: 10.1136/bmjopen-2023-075311, PMID: 37652587 PMC10476130

[50] Dierkes J Dahl H Lervaag-Welland N Sandnes K Sæle K Sekse I. Correction to: high rates of central obesity and sarcopenia in CKD irrespective of renal replacement therapy an observational cross-sectional study. BMC Nephrol. (2018) 19:375. doi: 10.1186/s12882-018-1149-1, PMID: 30583718 PMC6304759

[51] Xu B Guo Z Jiang B Zhang K Zhu W Lian X. Factors affecting sarcopenia in older patients with chronic diseases. Ann Palliat Med. (2022) 11:972–83. doi: 10.21037/apm-22-201, PMID: 35365027

