# Supplementary material for: Handgrip strength and body mass index exhibit good predictive value for sarcopenia in patients on peritoneal dialysis
Source: Front Nutr. 2024 Dec 13;11:1470669. doi: 10.3389/fnut.2024.1470669 (PMC11671354; doi:10.3389/fnut.2024.1470669)
Supplement: Supplementary file 1 [file Table_1.DOCX]

**Supplementary Table S1 Comparison of prevalence of PD sarcopenia in different age groups**

| **Age**  **(years)** | **Participants (n=625)** | | ***x*^2^** | ***p*** | **men (n=298)** | | ***x*^2^** | ***p*** | **women (n=327)** | | ***x*^2^** | ***p*** |
| --- | --- | --- | --- | --- | --- | --- | --- | --- | --- | --- | --- | --- |
|  | **non-sarcopenia**  **(n=538)** | **sarcopenia**  **(n=87)** |  |  | **non-sarcopenia**  **(n=255)** | **sarcopenia**  **(n=43)** |  |  | **non-sarcopenia**  **(n=283)** | **sarcopenia**  **(n=44)** |  |  |
| < 35 | 85 (15.80) | 14 (16.09) | 24.30 | < 0.05 | 46 (18.04) | 8 (18.60) | 16.02 | < 0.01 | 39 (13.78) | 6 (13.64) | 10.67 | 0.01 |
| 35-49 | 210 (39.03) | 20 (22.99) |  |  | 90 (35.29) | 8 (18.60) |  |  | 120 (42.40) | 12 (27.27) |  |  |
| 50-59 | 157 (29.18) | 20 (22.99) |  |  | 73 (28.63) | 9 (20.94) |  |  | 84 (29.68) | 11 (25.00) |  |  |
| 60-69 | 64 (11.90) | 21 (24.14) |  |  | 38 (14.90) | 11 (25.58) |  |  | 26 (9.19) | 10 (22.73) |  |  |
| ≥70 | 22 (4.09) | 12 (13.79) |  |  | 8 (3.14) | 7 (16.28) |  |  | 14 (4.95) | 5 (11.36) |  |  |

*x*^2^, Chi-Square test
