# Supplementary material for: Handgrip strength and body mass index exhibit good predictive value for sarcopenia in patients on peritoneal dialysis
Source: Front Nutr. 2024 Dec 13;11:1470669. doi: 10.3389/fnut.2024.1470669 (PMC11671354; doi:10.3389/fnut.2024.1470669)
Supplement: Supplementary file 2 [file Table_2.DOCX]

**Supplementary Table S2 Results of model diagnosis in male patients with sarcopenia and without sarcopenia**

| Model diagnosis | True diagnosis | |
| --- | --- | --- |
|  | sarcopenia | non-sarcopenia |
| sarcopenia | 34 | 25 |
| non-sarcopenia | 9 | 230 |
| positive predictive value = 34/(34+25)=0.5763 | | |
| negative predictive value = 230/(230+9)=0.9623 | | |
| Sensitivity = 34/(34+9)=79.07% | | |
| Specificity = 230/(230+25)=90.20% | | |
| Accuracy = (34+230)/(34+25+9+230）=88.59% | | |
