# Supplementary material for: Handgrip strength and body mass index exhibit good predictive value for sarcopenia in patients on peritoneal dialysis
Source: Front Nutr. 2024 Dec 13;11:1470669. doi: 10.3389/fnut.2024.1470669 (PMC11671354; doi:10.3389/fnut.2024.1470669)
Supplement: Supplementary file 3 [file Table_3.DOCX]

**Supplementary Table S3 Results of model diagnosis in female patients with sarcopenia and without sarcopenia**

| Model diagnosis | True diagnosis | |
| --- | --- | --- |
|  | sarcopenia | non-sarcopenia |
| sarcopenia | 39 | 42 |
| non-sarcopenia | 5 | 241 |
| Positive predictive value = =39/(39+42) = 0.4815 | | |
| Negative predictive value = 241/(241+5) = 0.9797 | | |
| Sensitivity = 39/(39+5) = 88.64% | | |
| Specificity = 241/(241+42) = 85.16% | | |
| Accuracy = (39+241)/(39+42+5+241）= 85.63% | | |
